# Use of Rasch person-item maps to validate a theoretical model for measuring Attitudes toward Sexual Behaviors

**DOI:** 10.1371/journal.pone.0202551

**Published:** 2018-08-23

**Authors:** Andrea Blanc, Antonio J. Rojas

**Affiliations:** Department of Psychology, University of Almeria, Almeria, Spain; Qazvin University of Medical Sciences, ISLAMIC REPUBLIC OF IRAN

## Abstract

In general, the Attitudes toward Sexual Behaviors (ASB) or Sexual Attitudes measures have not been focused in the conjoint measurement of persons and items in the same construct, and have not reflected the new sexual behaviors neither the context where sexual behaviors take place. The purpose of this study was to provide evidence of the validity of a theoretical model for the measurement of ASB using the person-item map provided by a Rasch model. The theoretical model explicitly defines the ASB construct and makes a proposal where the items are intensity-ordered. It also considers the context in which sexual behaviors take place and their new ways of expression. ASB measurement was applied to two samples of 300 and 584 young people aged 18 to 30. Content of the ASB test administered includes the operative definition proposed. The results showed a good fit of the data to the model, and adequate measurement accuracy. The person-item maps showed that the items are distributed according to the theoretical model proposed in both samples. The easiest items for participants to endorse are those reflecting frequent dyadic sexual behaviors with a steady partner, and the hardest items for participants to endorse are those reflecting sexual behaviors via Information and Communication Technologies. The obtained results permit to conclude that there is favorable validity evidence for the theoretical model proposed for measuring ASB in heterosexual young people.

## Introduction

Many instruments have been designed to measure different facets of sexual attitudes, such as the Sexual Opinion Survey [1], based on the erotophobia-erotophilia concept or Trueblood Sexual Attitudes Questionnaire [2], based on the conservatism-liberalism concept. However, few have been developed exclusively to measure Attitudes toward Sexual Behaviors (ASB) [3]. Moreover, neither sexual attitude instruments nor ASB instruments usually reflect the context where the specific sexual behaviors take place (e.g., casual relationship) in the content of their items, nor given the time when they were published [4], do they include the new and emerging sexual behaviors (e.g., sexting). Additionally, the measurement model usually used in the sexual attitude instruments is the Classical Test Theory, only focused on person measures and where each item is equally weighted in the estimation of construct. For these reasons, to develop a model for the measurement of ASB which considers the context where sexual behaviors occur and the new sexual behaviors, and which enables conjoint measurement of persons and items in the same construct is essential (each item is not equally weighted in the estimation of construct).

First, to establish a theoretical framework providing support for an operative ASB definition useful for measuring it, the instrument’s content must be specified. On the one hand, in the case of ASB, this content must collect the main sexual behaviors. In general, research focused on sexual behaviors has undergone changes brought about by the socio-historical moment in which studies were done. In the beginning, much research in the area of sexuality concentrated on sexual intercourse in general and the vaginal intercourse was the most considerate sexual behavior [5, 6]. This is because for centuries, the only acceptable sexual behavior was the vaginal intercourse within the context of marriage and for the purpose of procreation [7]. Later, sexual activity started to expand and research began to differentiate specific sexual behaviors such as kissing and caressing, vaginal coitus, masturbation, oral sex, and anal sex [8]. Oliver and Hyde [9] in a meta-analysis of gender differences in sexuality, mention different specific sexual behaviors like kissing, petting, intercourse, masturbation, oral sex, etc. From 1943 to 1999 people´s sexual attitudes and behavior changed substantially in the United States [10]. For instance, young women and men became more sexually active (e.g., age at first intercourse decreased from 19 to 15 years among young women), attitudes toward premarital intercourse became more permissive and feelings of sexual guilt decreased over time [10].

Subsequently, Petersen and Hyde [11] in a meta-analytic review of research on gender differences in sexuality (from 1993 to 2007) added sexual behaviors such as anal sex, pornography use, cybersex, casual sex, and same-gender sexual behavior (among others), and eliminated kissing (because few studies evaluated the incidence of kissing in the last two decades). These new sexual behaviors show that at the present time sexual activity is not morally restricted to traditional heterosexual marriage: occurs between unmarried romantic partners, casual partners, and between people of different sexual orientations, and is not purely focused on procreation [7]. Moreover, Twenge, Sherman, and Wells [12] demonstrate that U.S. adults in 2000–2012 (vs. the 1970 and 1989s) had more sexual partners, were more probably to have had sex with casual partners, and were more accepting of non-marital sex.

With respect to the development of a theoretical model, the ASB instrument’s content must collect the wide range of sexual behaviors hierarchically ordered. For this, the classifications on sexual behaviors are very important. Sexual behaviors may be divided basically by how they are performed [7] and their frequency [13, 14]. Sexual behaviors may be done in solitary, with a partner (dyadic) or with more than one person at the same time. They may also be very frequent (e.g., caressing and coitus), frequent (e.g., oral sex and masturbation) or infrequent (e.g., anal sex and group sex). Studies that have divided sexual behaviors according to their frequency have included mostly people with heterosexual orientation [14]. However, results on the frequency in specific sexual behaviors in non-heterosexual people are different [15].

Solitary sexual behaviors studied most and which should be included in a theoretical model for measuring ASB are solitary masturbation [16, 17] and sexual fantasies [18, 19]. Solitary masturbation may be accompanied by other sexual behaviors which should also be included, such as the use of erotic material. Although erotic material may be used by couples, studies show they are used more in solitary [20, 21].

Among the dyadic sexual behaviors studied most and which should be reflected in a model for measuring ASB are caressing [22], penile-vaginal intercourse [23, 24], oral sex [25, 26], mutual masturbation [14, 27] and anal sex [24, 28]. In addition to these sexual behaviors, with the development of the new Information and Communication Technologies (ICT), new sexual behaviors, such as cybersex [29] and sexting [30] have emerged. Finally, the sexual behaviors performed with a partner can also be done with more than one person at the same time. This type of sexual relations called threesome [31] and group sex [32] should also be included.

In addition to specific sexual behaviors, the context in which they take place is also important. Chambers [33] found that a large percentage of people feel more comfortable when certain sexual behaviors, such as oral sex, are done within a steady relationship than when they are done with a casual partner. Similarly, cybersex has been found to be more frequent with a steady partner than a casual partner, like someone they do not know well or at all [34], and sexting is more common between steady partners than uncommitted singles [35]. Looking at the context of sexual behaviors, solitary sexual behaviors may be done when a person has a partner or not, but is less frequent when a person has a steady partner [17]. Dyadic sexual behaviors may mainly take place with a steady partner or with a casual partner [24].

**Fig 1:**
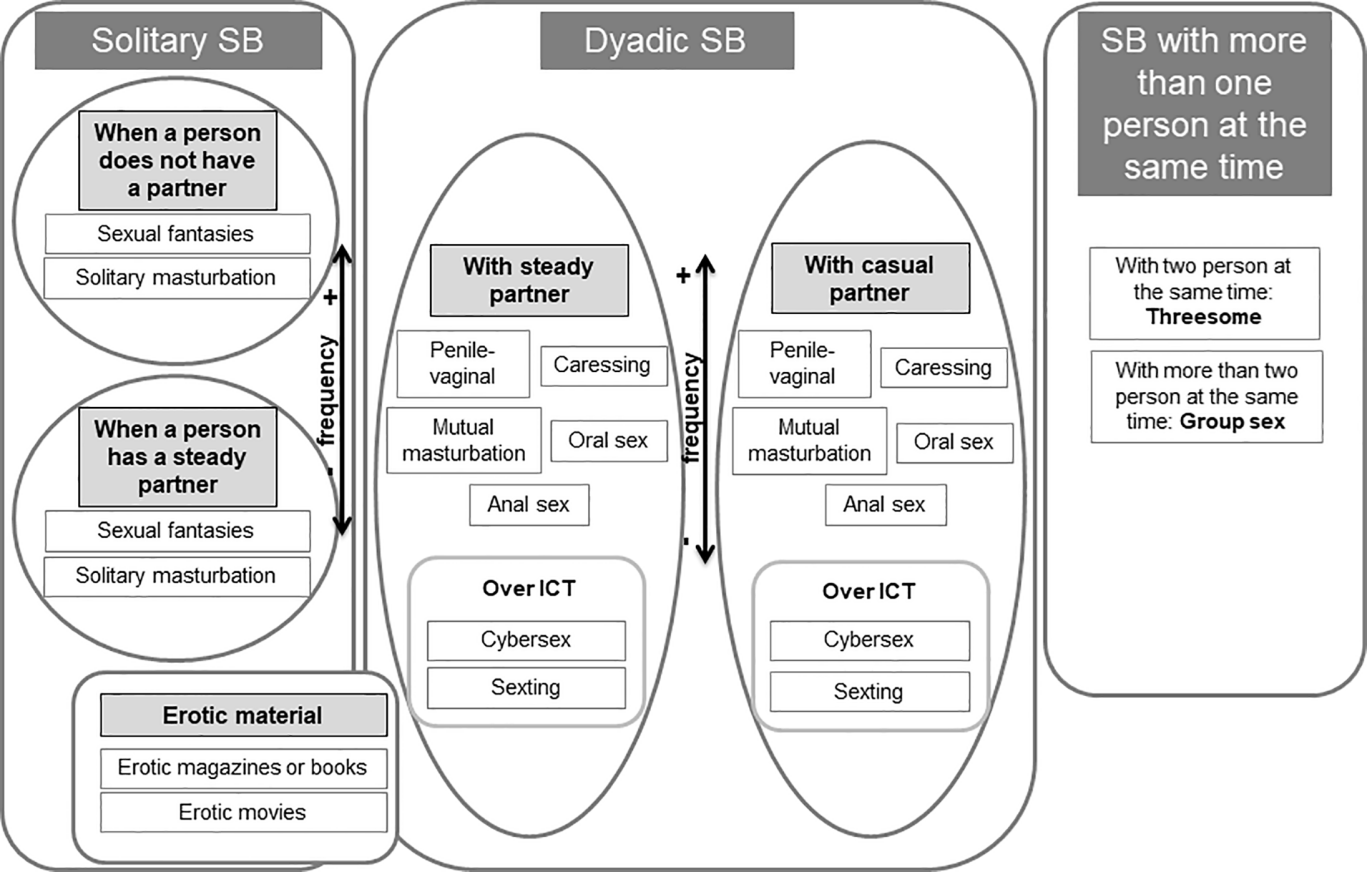
Theoretical framework of the Attitudes toward Sexual Behaviors. *Note*. ICT = Information and Communication Technologies; SB = Sexual Behaviors.

Therefore, this study was based on a new theoretical model for measuring ASB which includes solitary sexual behaviors, dyadic sexual behaviors and sexual behaviors with more than one person at the same time, and in different contexts. For solitary sexual behaviors when one has a partner or not and for dyadic sexual behaviors, with a steady or casual partner. Furthermore, this model considers very frequent dyadic sexual behaviors in heterosexual people (caressing, penile-vaginal intercourse, mutual masturbation, and oral sex) and an infrequent dyadic sexual behavior in heterosexual people (anal sex) and includes new sexual behaviors, such as cybersex and sexting. The model also contains the use of erotic material like erotic magazine or books, and erotic movies. Finally, it includes the sexual relations with more than one person at the same time such as threesome and group sex. The new theoretical model for measuring ASB is shown in the Fig 1

Although attitude has had many different definitions, [36–38], they all agree that attitude may be inferred from evaluations of objects in a dimension that varies from positive to negative [39]. An attitude is a psychological tendency expressed by the evaluation of a particular attitudinal object with some degree of favor or disfavor [40]. In this study, the attitudinal object, which is the stimulus that elicits the response, are sexual behaviors. But on an attitudinal continuum of favor or disfavor, not all sexual behaviors should have the same value [41]. Although in the context of sexual attitudes, most instruments is based on the Classical Test Theory, where each item is equally weighted in the estimation of construct, this proposal makes a conjoint measurement of persons and items based on an Item Response Theory model. The novelty of this model is that it enables the items to have different difficulties in the estimation of construct (person’s ASB): e.g., an item reflecting penile-vaginal intercourse with a steady partner may be easier for participants to endorse than an item reflecting cybersex with a casual partner.

In a stimulus-centered attitude scaling, Blanc and Rojas [42] showed that items reflecting frequent sexual behaviors (penile-vaginal intercourse, oral sex, and caresses) are easier for participants to endorse than items reflecting infrequent sexual behaviors (anal sex, group sex, and cybersex). In this study was also found that frequent and infrequent sexual behaviors were closer in people with casual partners than in people with steady partners in the continuum, evidencing the need to contextualize the sexual behaviors.

In general, the psychometric theory predominant in developing instruments for measuring sexual attitudes is the Classical Test Theory. In this psychometric model, mainly focused on reliability, the person total scores have been obtained by assigning the same score to each item within the instrument [43]. The Classical Test Theory assumes that each item usually contribute with the same amount in the estimation of construct and only locates persons in the continuum (uses a person-centered scaling). The Item Response Theory is an alternative to the Classical Test Theory. Among the models based on the Item Response Theory are Rasch models. Rasch models offer relevant advantages over the Classical Test Theory because resolve the double invariance problem: person measures are independent of the instrument used, and, the estimates of the items and test properties are independent of the sample of individuals used. Moreover, while there is an only one standard error of measurement for the whole test in the Classic Test Theory, in Rasch models there are standard errors of measurement associated with each level of the construct.

Rasch models also allow conjoint measurement of persons and items in the same construct and can estimate the extent to which each item measures the ability of the people. This is, each item has its own contribution (item parameter or item difficulty) for the estimation of construct measured. Rasch models allow the elaboration of unidimensional scales for latent variables where the probability of the person´s response to an item is a logistic function determined by the person’s ability (person parameter) and item difficulty (item parameter). These models transform the raw scores of persons and items on an interval scale (the same metric for persons and items) called logits (or log-odds units), with a mean of 0 and standard deviation of 1 [44]. This measurement scale enables to interpret the differences in measurement of persons and items in a similar way throughout the entire construct.

Furthermore, Rasch models can be used as a construct validation tool because it permits to build a hypothetical unidimensional line along which items are hierarchically located according to their difficulty and persons by their abilities [45]. They can “map” items and persons, that is, find out the location of the items on the continuum along with distribution of the person measures. Observation of this map and comparison to the theoretical proposals the scale is based on (which define how the items should be located on the continuum) provides a powerful source of evidence of validation of the theoretical models. Among the Rasch models, the *rating scale model* [44] is a specific polytomous model applicable to measuring attitudes where all the items have the same graduated response scales (e.g., Likert-type items). In the case of ASB measurement, by applying this model, the items referring to sexual behaviors can be mapped if the data fit to the model.

The purpose of this research was to find evidence of validity of a new theoretical model proposed for the measurement of ASB using the person-item map of a rating scale model. It was expected that the items reflecting sexual behaviors would be grouped and distributed according to the following hypotheses: a) the items reflecting dyadic sexual behaviors with a steady partner should have a lower value on the scale (continuum) than the items reflecting dyadic sexual behaviors with a casual partner (the first ones should be easier for participants to endorse than the second ones), b) items reflecting very frequent dyadic sexual behaviors should have a lower value on the scale than the items reflecting infrequent dyadic sexual behaviors, c) the items reflecting solitary sexual behaviors when a person does not have a partner should have a lower value on the scale than the items reflecting solitary sexual behaviors when a person has a partner, d) the items reflecting solitary sexual behaviors (solitary masturbation and sexual fantasies) are going to be located close to each other on the continuum along with the items reflecting the use of erotic material (they should have similar difficulties for participants to endorse), and e) the items reflecting infrequent sexual behaviors (anal sex, threesome and group sex) and sexual behaviors via ICT should have the highest values on the scale (they should be the hardest items for participants to endorse).

## Method

In this study, the instrument was applied to two independent samples, each with different item response choices. This was done to replicate the distribution of the location of the items on the map and see if it matched to the theoretical proposal for ASB measurement.

### Participants

The first sample included 300 young participants ranging in age from 18 to 30 years, selected by quota sampling by sex. The second sample consisted of 584 young participants ranging in age from 18 to 30 years, also selected by quota sampling by sex. The sociodemographic and sexual characteristics of both samples are shown in Table 1. Because most participants had a heterosexual orientation and the orientation might influence in the results, only heterosexual people were included in the analysis.

**Table 1:** Sociodemographic and sexual characteristics of both samples.

Variables		First Sample	Second Sample
Sex	Women	150 (50%)	292 (50%)
	Men	150 (50%)	292 (50%)
Age	M	21.56	22.20
	SD	2.73	3.27
Level of education	Primary or high school	174 (58.0%)	404 (69.2%)
	University studies	126 (42.0%)	182 (30.1%)
Partner	Steady partner	154 (51.3%)	281 (48.1%)
	Casual partner	29 (9.7%)	55 (9.4%)
	No partner	117 (39.0%)	248 (42.5%)
Sexual orientation	Heterosexual	291 (97.0%)	536 (91.8%)
	Homosexual	4 (1.3%)	25 (4.2%)
	Bisexual	4 (1.3%)	23 (3.9%)
	Asexual	1 (0.3%)	0 (0.0%)

### Instrument

#### Test of Attitudes toward Sexual Behaviors (TASB)

This test (Appendix A) was designed to measure attitudes toward specific sexual behaviors in different contexts, understanding ASB as the psychological tendency expressed by evaluation of sexual behaviors with some degree of favor or disfavor. The TASB is made up of 22 items, where each item refers to performance of a sexual behavior. Dyadic sexual behaviors included are caressing, penile-vaginal intercourse, mutual masturbation, oral sex, anal sex, cybersex, and sexting (14 items, 7 items referring to a steady partner and 7 items with a casual partner), the solitary sexual behavior were solitary masturbation and sexual fantasies (4 items, 2 items reflecting when a person has a partner and 2 items when a person does not have a partner), and the sexual behavior with more than one person at the same time were threesome and group sex (2 items). Finally, the test includes 2 items referring to the use of erotic material (erotic magazines and books and erotic movies). All the items have a five-choice Likert-type format (from -2 very negative to +2 very positive). The higher the score, the more positive the ASB is.

For the second sample, since the analysis of the functioning of TASB item response choices showed that five could be many categories, it was decided to include only three item choices. The participants had to evaluate the sexual behavior with only three item response choices: negative, neither negative nor positive, or positive. The higher the score, the more positive the ASB is.

#### Sociodemographic items

Questions about sex, age, level of education, relationship status, and sexual orientation were included.

### Procedure

In the first sample, the test was administered individually in computerized format at the facilities of a university of Spain outside of class hours. The study was disseminated in that university. The first block presented dyadic sexual behaviors with a casual partner, the second dyadic sexual behaviors with a steady partner and in the third block, the rest of the sexual behaviors. Participants were given a gratification of 5 Euros.

In the second sample, the test was administered on the Google Forms platform. Students from another university of Spain were sent a link so they could answer individually and spread it among their acquaintances. First, students were informed about the study during class hours and they had a week to participate. Later, they spread the link to their acquaintances. Only student acquaintances were included in this sample. The whole set of items was presented in a manner similar to the first sample. Participants were not given any gratification.

All participants signed an Informed Consent Form before they completed the questionnaire. The topic of the study, the inclusion criteria (e.g., being of legal age), the anonymity, the voluntariness, and the rights of the participants were contained in that consent form. The study was also approved by the Bioethics Committee on Human Research of the University of Almeria.

### Data analysis

The psychometric model applied in both samples was the rating scale model [44], a polytomous Rasch model. The rating scale model considers the probability of the person answering one of the response categories of an item as a logistic function determined by the person’s ability (known as person’s parameter which allows locating persons on the unidimensional continuum, in this case according to his/her level of ABS construct) and the item difficulty (known as item’s parameter, which locate the items on the unidimensional continuum, in this case according to how easy/difficult it is to evaluate favorably a sexual behavior).

First, the existence of atypical values resulting from two cases in the first sample and ten cases in the second sample were analyzed and eliminated. Later, fit of data to the rating scale model was checked by residual analysis (differences between the values expected by the model and data observed) for the overall fit (items and persons), as well as fit for each of the items. The analysis concentrated on the mean square fit statistics analyzed based on two indices, the infit (sensitive to unexpected response patterns to items near the person measure level) and outfit (sensitive to unexpected response patterns to items far from the person measure level). These statistics are appropriate and the data fit to the model when they vary from 0.4 to 1.6.

To analyze dimensionality, the Principle Components Analysis (PCA) of standardized residuals was calculated. If the variance explained by measures (item parameters, person parameters and rating scale structure) is greater than 60%, and the unexplained variance in first contrast is less than 5.0%, the assumption of unidimensionality is fulfilled. To analyze local independence, the residual correlations between items were calculated. Residual are those parts of the data not explained by the Rasch model [46]. High correlation of residuals for two items shows that those items may be locally dependent [46]. When these correlations are around .70 the assumption of local independence is not met [47].

Having checked fit of data to the model, person reliability and the separation indices for persons and items were analyzed. Reliability in the rating scale model is conceived as the percentage of answers observed that can be reproduced and estimated for both items and persons by the separation indices. Reliability varies from 0 to 1.00. Person reliability is equivalent to the test reliability reported by Classical Test Theory [46]. The separation index for persons is used to classify people and represent the number of different strata the instrument can detect in the sample. When this index is less than two (and person reliability < .80), it shows the instrument cannot differentiate between persons with high and low scores and more item may be needed. The item separation index is useful for differentiating the order for locating the items on the unidimensional continuum (item hierarchy). This order is important for the amount of construct they must have according to the specifications of the test content to be compared to the order estimated from the data. When this index is below three it implies that persons in the sample are insufficient to confirm the order of the items (item difficulty hierarchy) and the test does not have items with high, low and medium difficulty [46].

Measurement accuracy was analyzed graphically using standard error of measurement associated with each ability level of the construct. Finally, a person-item map was obtained to locate the distribution of the items indicative of each sexual behavior and the person measures on the continuum and to obtain validity evidence of the theoretical model proposed. All analyses were done using WINSTEPS version 3.63.2 software [46].

## Results

### Fit of data to the model

Table 2 shows the mean square residual statistics for both infit (IMNSQ) and outfit (OMNSQ) for both samples. For the overall person fit, the infit and oufit mean-square statistics are 1.04 and 1.00 in the first sample, and are 1.02 and 0.99 in the second sample, respectively. For the overall item fit, the infit and oufit mean-square statistics are 0.51 and 0.53 in the first sample, and are 0.45 and 0.91 in the second sample, respectively. Infit and outfit mean-square statistics for items applied to the first sample vary from 0.67 to 1.57. Fit statistics of each item applied to the second sample varying from 0.52 to 1.49. Therefore, all the fit statistics are appropriate and compatible with the fit of data to the model.

**Table 2:** Mean square fit statistics for the TASB for each sample.

	Measure	Error	IMNSQ	OMNSQ	Item-total Correlation
**Person M**	0.70 (1.33)	.28 (.47)	1.04 (1.02)	1.00 (0.99)	
**Person SD**	0.87 (1.29)	.08 (.13)	0.51 (0.45)	0.53 (0.91)	
**Item M**	0.00 (0.00)	.08 (.11)	1.03 (0.99)	1.00 (0.99)	
**Item SD**	1.28 (1.78)	.03 (.06)	0.18 (0.18)	0.22 (0.24)	
I1.Caressing CP	-0.34 (-0.40)	.07 (.09)	0.81 (0.89)	0.78 (0.98)	.59 (.52)
I2. Penile-vaginal intercourse CP	-0.21 (-0.26)	.07 (.08)	0.96 (0.95)	0.90 (0.85)	.61 (.55)
I3.Mutual masturbation CP	0.04 (-0.24)	.06 (.08)	0.72 (0.84)	0.71 (0.88)	.68 (.58)
I4.Oral sex CP	0.38 (0.84)	.06 (.07)	1.03 (1.04)	1.04 (0.98)	.64 (.61)
I5.Anal sex CP	1.39 (2.05)	.06 (.07)	0.96 (0.98)	1.03 (1.00)	.61 (.67)
I6.Sexting CP	1.81 (2.61)	.07 (.08)	1.37 (1.20)	1.41 (1.25)	.50 (.61)
I7.Cybersex CP	2.03 (3.02)	.07 (.09)	0.93 (1.02)	0.92 (0.97)	.60 (.66)
I8.Caressing SP	-2.67 (-3.65)	.15 (.28)	1.04 (0.98)	0.85 (0.65)	.29 (.16)
I9. Penile-vaginal intercourse SP	-3.14 (-3.65)	.19 (.28)	1.15 (0.98)	0.85 (0.72)	.26 (.16)
I10. Mutual masturbation SP	-1.51 (-2.67)	.10 (.18)	1.28 (0.95)	1.06 (0.52)	.43 (.30)
I11. Oral sex SP	-1.39 (-1.95)	.09 (.14)	1.11 (1.03)	0.90 (1.14)	.55 (.34)
I12. Anal sex SP	0.51 (0.51)	.06 (.07)	1.19 (1.31)	1.23 (1.24)	.59 (.53)
I13. Sexting SP	0.76 (0.81)	.06 (.07)	1.56 (1.47)	1.57 (1.49)	.47 (.49)
I14. Cybersex CP	1.07 (1.44)	.06 (.07)	1.37 (1.22)	1.33 (1.30)	.57 (.59)
I15.Solitary masturbation NP	-0.57 (-0.84)	.07 (.09)	0.81 (0.77)	0.84 (0.71)	.61 (.52)
I16. Solitary masturbation SP	0.09 (0.02)	.06 (.08)	0.95 (0.88)	0.97 (1.07)	.59 (.56)
I17.Sexual fantasies NP	-0.68 (-1.16)	.08 (.10)	0.85 (0.73)	0.87 (0.94)	.49 (.47)
I18. Sexual fantasies SP	-0.03 (-0.27)	.07 (.08)	1.01 (1.09)	1.01 (1.44)	.50 (.45)
I19.Erotic magazines or books	0.34 (0.24)	.06 (.08)	0.74 (0.73)	0.83 (0.84)	.60 (.59)
I20.Erotic movies	0.18 (0.25)	.06 (.08)	0.67 (0.77)	0.67 (0.86)	.67 (.60)
I21.Threesome	0.70 (1.26)	.06 (.07)	1.00 (1.00)	1.02 (1.01)	.62 (.63)
I22.Group sex	1.26 (2.03)	.06 (.07)	1.06 (0.90)	1.14 (0.91)	.56 (.67)

*Note*. Values for sample 2 are in parenthesis; CP = casual partner; SP = steady partner; NP = no partner.

The item measures (their level of difficulty) in logits for the first sample vary from -3.14 to 2.03 and the item-total correlations from .26 to .68. For the second sample, the measures of the items in logits vary from -3.65 to 3.02 and the item-total correlations from .16 to .67. In this last sample, the item measures in logits show a wider range.

### Unidimensionality and local independence

In the first sample, the PCA of the standardized residuals shows that the variance explained by measures is 86.4%, and the unexplained variance in first contrast is 2.3%. In the second sample, the PCA of the standardized residuals shows that the variance explained by measures is 92.2%, and the unexplained variance in first contrast is 1.0%. Thus, the measure can be considered unidimensional.

In both sample, none of the standardized residual correlations are higher than .70. Moreover, of the 231 residual correlations only six (2.6%) in the first sample and four (1.7%) in the second sample are higher than .50. Therefore, the largest standardized residual correlations are limited and the local independence can be assumed.

### Separation indices and reliability

In the first sample, the separation index for persons is 2.80 and person reliability is .89. This separation index shows that the instrument is sufficiently sensitive to distinguish between almost three groups of persons (with negative, neutral and positive attitudes toward sexual behaviors). The separation index for items is 14.46. This separation index shows that the sample is adequate for differentiating items with different difficulty levels on the scale and comparing the data with the specifications of the test content proposed (construct validity). In the second sample, the separation indices for persons and items are 2.47 and 14.37, and person reliability is .85. Thus, separation indices for persons and items and person reliability are adequate in both samples.

### Measurement accuracy for each level of the ASB construct

**Fig 2:**
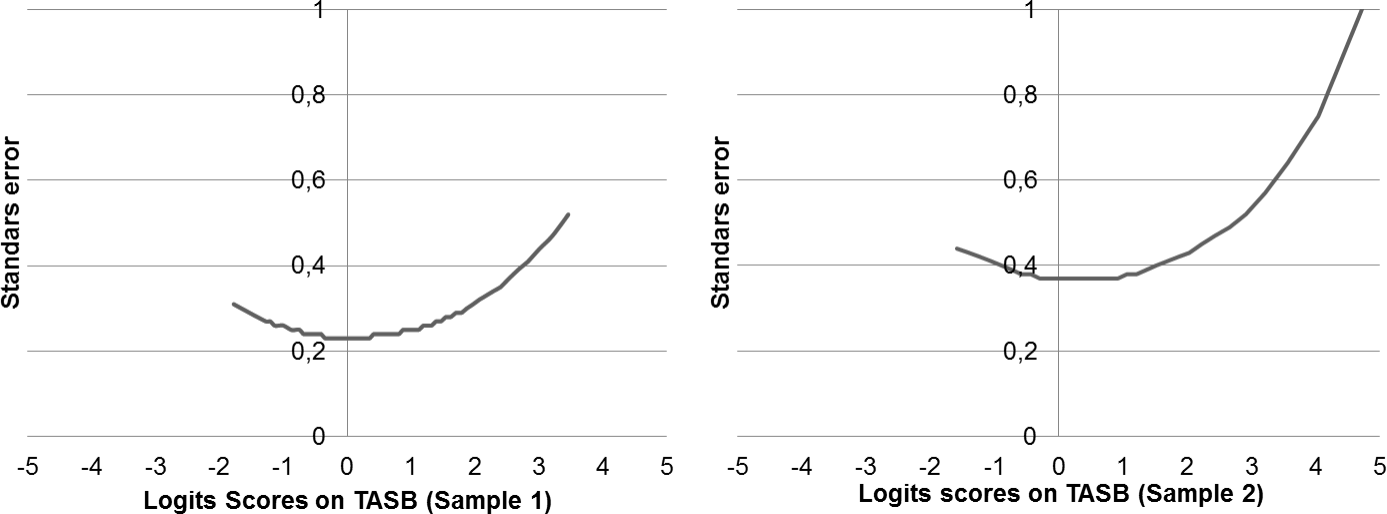
Standard errors of measurement for each ability level in the TASB in both samples.

TASB accuracy in terms of the standard error of measurement for each level of the construct in both samples appears in Fig 2. In both samples, the TASB is more accurate in the middle of the continuum than at the extremes (i.e., measurement error is higher in persons with extreme scores). Moreover, if the two error curves are compared, the measurement is better, in terms of precision, in the first sample than in the second sample, especially in the middle of the continuum. Whereas in the first sample, where the measurement has five response categories, the standard error of measurement in the middle is almost 0.2, in the second sample, where the scale has three response categories, the standard error is nearly 0.4. This result demonstrates that the TASB with five response categories is better, in terms of precision, than with three response categories.

### Validity evidence of the theoretical proposal based on the person-item map

**Fig 3:**
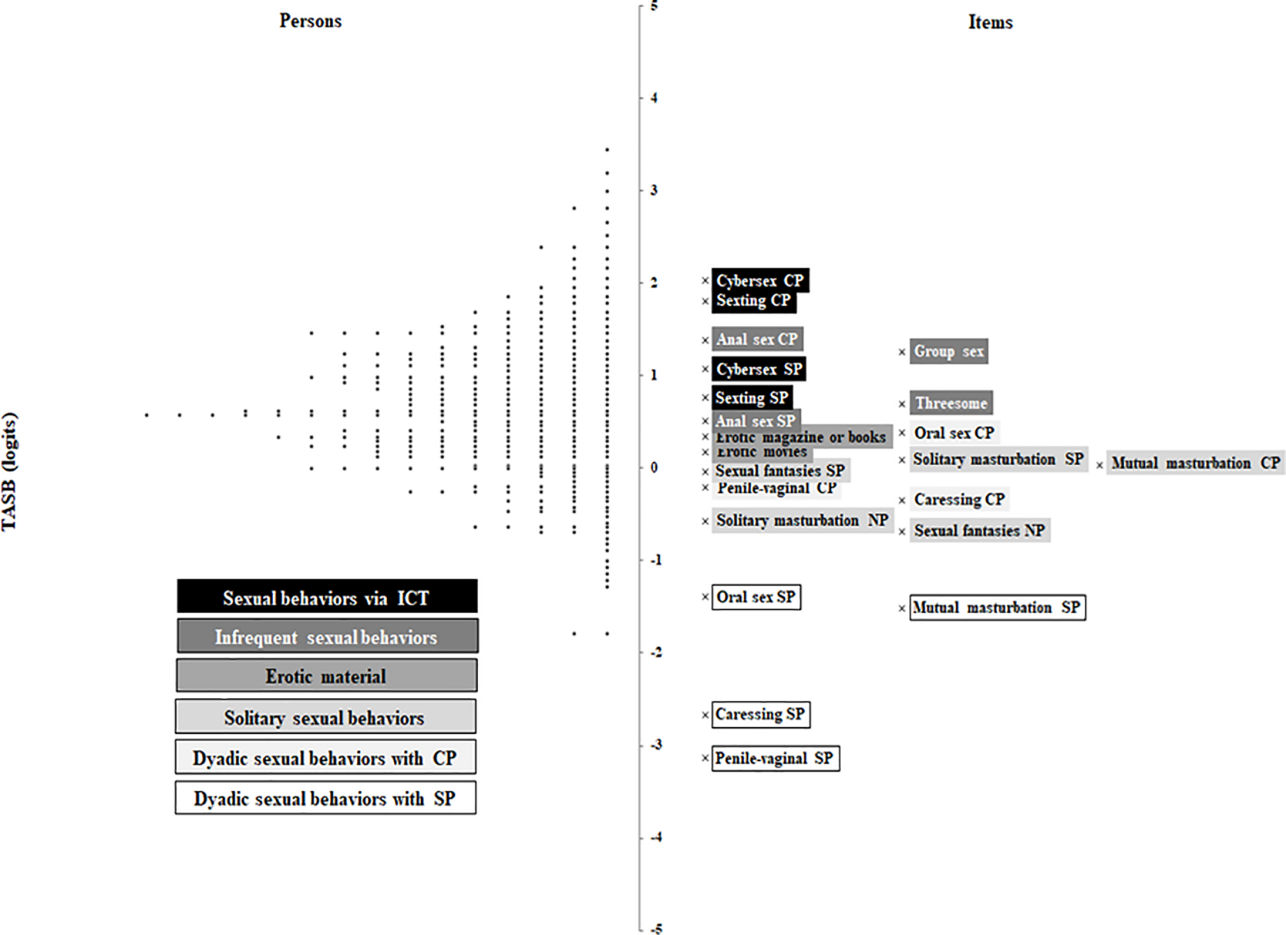
Item-person map of the sample 1. *Note*. **· =** one person; CP = casual partner; SP = steady partner; NP = no partner; ICT = Information and Communication Technologies.

The person-item map [48] shows the conjoint distribution of the person measures and the location of the items on the same continuum measured. The items are on the right of the map and persons are on the left. The hardest items for participants to endorse (those which have the highest logit measures) are located at the top of the map, and the easiest items for participants to endorse (have the lowest logit measures) are at the bottom. In the first sample (Fig 3), the easiest item for participants to endorse is “penile-vaginal sexual intercourse with a steady partner” (-3.14 logits), and the hardest item is “sex over the internet (cybersex) with a casual partner” (2.03 logits). Fig 3 shows how the items referring to very frequent dyadic sexual behaviors (caressing and penile-vaginal sexual intercourse) and frequent dyadic sexual behaviors (oral sex and mutual masturbation) with a steady partner are at the bottom. In the middle of the map are the items referring to: solitary sexual behaviors (solitary masturbation and sexual fantasies), the use of erotic material (erotic magazines and books and erotic movies) and, very frequent and frequent dyadic sexual behaviors with a casual partner. Finally, at the top are the items referring to infrequent sexual behaviors (threesome, group sex and anal sex), as well as sexual behaviors via ICT (cybersex and sexting).

In general, the items referring to dyadic sexual behaviors with a steady partner are easier for participants to endorse than the items referring to dyadic sexual behaviors with a casual partner. The items referring to solitary sexual behaviors when a persona does not have a partner are easier for participants to endorse than the items referring to solitary sexual behaviors when a person has a partner. The items referring to erotic material are near on the map of the latter.

Each black dot “●” represents a person measure. Although they are distributed throughout the continuum, the highest percentage is located in the higher middle of the continuum and seems to form a N distribution of the measure. The persons with have low scores in the construct are located at the bottom of the map and they are expected to evaluate the items above them (with more amount on the ASB construct or more score) negatively, such as cybersex and sexting. For instance, it is probable that a person with a logit score of 2.00 evaluated all items negatively except “penile-vaginal sexual intercourse with a steady partner” (-3.14 logit) and “caressing any intimate part of the body of a steady partner” (-2.67 logit). On the contrary, persons with very high scores are at the top of the map and it is probably that they evaluated both items at the bottom (e.g., oral sex with steady partner) and those at the top (e.g., group sex) positively. For instance, a person with a logit score of 3.00 (with positive ASB) it is probable that evaluated all items positively.

**Fig 4:**
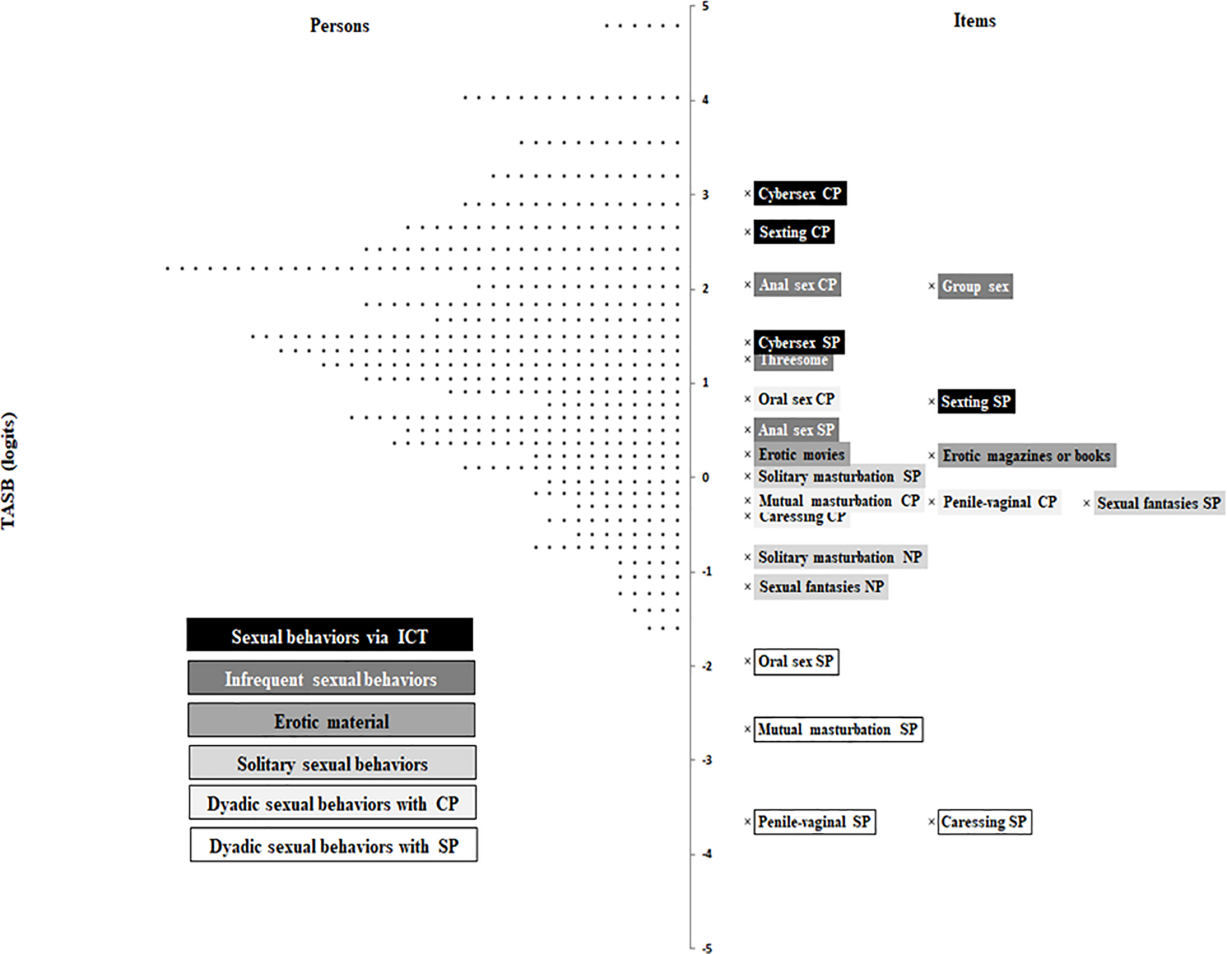
Item-person map of the sample 2. *Note*. **· =** one person; CP = casual partner; SP = steady partner; NP = no partner; ICT = Information and Communication Technologies.

In the second sample (Fig 4), the easiest items for participants to endorse are “penile-vaginal sexual intercourse with a steady partner” and “caressing any intimate area of the body of a casual partner” (-3.65 logits), and the hardest item is “sex over the internet (cybersex) with a casual partner” (3.02 logits). These results are similar to the first sample. Grouping and distribution of the items on the map is also similar. At the bottom are the items that refer to very frequent and frequent dyadic sexual behaviors with steady partner. In the middle, items referring to solitary sexual behaviors, the use of erotic material and very frequent and frequent dyadic sexual behaviors with a casual partner. At the top are the items to referring to infrequent sexual behaviors and sexual behaviors via ICT. In this sample, the distribution of person measures is located at slightly higher values than in the first sample.

## Discussion

The aim of this study was to provide evidence of validity of a theoretical model for the measurement of ASB using a person-item map of a Rasch model, such as the rating scale model. Validity evidence of this theoretical model was obtained by observing the location of the item in this map and comparing them with the theoretical model, which define how they should be located on the continuum. Previously, the fit of the data to the model, unidimensionality and local independence, and the measurement accuracy were analyzed. The overall person fit, overall item fit, and fit of each item were adequate. Unidimensionality and local independence can be assumed. Likewise, separation indices, reliability, and measurement accuracy for each level of the ASB construct were also adequate. Although, the TASB with five response categories (first sample) was more precise than the TASB with three response categories (second sample).

Once goodness of fit of the data to the model and adequate measurement accuracy had been shown, a conjoint person-item map was made for the two independent samples using the same scale but with different item response choices. In both samples, the easiest item for participants to endorse was “penile-vaginal sexual intercourse with a steady partner”. In the second sample, the easiest item was also “caressing any intimate area of the body of a casual partner”. This finding is coherent with the theoretical proposal because for many years the only sexual behavior socially accepted was penile-vaginal intercourse in marriage. On the contrary, the hardest item for participants to endorse in both studies was “sex over the internet (cybersex) with a casual partner”. In general, the results show that the items referring to dyadic sexual behaviors with a steady partner are easier for participants to endorse than the items referring to dyadic sexual behaviors with a casual partner (hypothesis a). This is analogous to what has been found in previous studies, where most people feel more comfortable with certain sexual behaviors with a steady partner than with a casual one [33]. It is also related to sexting and cybersex being more frequent among persons with steady relationships [34, 35, 49].

The easiest items referring to dyadic sexual behaviors are those including penile-vaginal sexual intercourse and caressing, and the hardest items referring to dyadic sexual behaviors are those including anal sex, sexting and cybersex (Hypothesis b). This result agrees with the findings of Blanc and Rojas [42] described earlier. The items reflecting frequent sexual behaviors are easier for participants to endorse than the items reflecting infrequent sexual behaviors.

On the other hand, the items referring to dyadic sexual behaviors with a casual partner are closer than the items referring to dyadic sexual behaviors with a steady partner. This is coherent with that Blanc and Rojas [42] found in their study, the frequent and infrequent sexual behaviors were closer in people who have a casual relationship than in people who have a steady relationship in the continuum.

The items referring to solitary sexual behaviors when a person does not have a partner (as expected) are easier than the items referring to solitary sexual behaviors when a person has a partner (Hypothesis c). These differences coincide with stronger acceptance of masturbation when a person does not have a partner than when has a partner [50]. Moreover, as expected the items referring to use of erotic material (erotic magazines and books and erotic movies) are near solitary sexual behaviors when a person has a partner on the map (Hypothesis d), and therefore, have similar difficulties for participants to endorse. This finding is consistent with other studies where the 83% of young men and the 55% of young women informed masturbating while viewing pornography [20]. The location of items referring to the use of erotic material shows that the use of erotic magazines and books and erotic movies are evaluated more negatively than frequent dyadic sexual behaviors, but more positively than infrequent sexual behaviors and sexual behaviors via ICT.

As expected, the hardest items for participants to endorse are those that include infrequent sexual behaviors (threesome, group sex and anal sex) and sexual behaviors via ICT (cybersex and sexting; Hypothesis e). On the one hand, many people may evaluate the sexual relations with more than one person at the same time negatively because this kind of relationship is different from the traditional relationships formed only by two people. In addition, the group sex may be harder than the threesome because it involves even more people. On the other hand, many people may evaluate sexual behaviors via ICT negatively mainly for two reasons: newness and risks. These behaviors are the newest and many people are still unfamiliar with them. They are also behaviors done by internet, and people who do them could lose control of the information they send, be deceived or blackmailed (e.g., sextortion) [51].

It may be observed on the map that in both samples, the items that make up the TASB are distributed suitably over the whole continuum to cover most of the sample in the study. That is, the results, using the Rasch person-item maps, show favorable evidence of validity to use the theoretical model for measuring Attitudes toward Sexual Behaviors in young people from 18 to 30 years of age.

Among the limitations of this study are the use of non-random samples, in which quotas were used for selecting the participants, and the limited number of non-heterosexual participants that were not included in the data analysis. Future studies should replicate this theoretical model proposed, not only in random samples, but also applied to people with a wider age range, from other cultures, and other sexual orientation. In addition, item invariance across different groups (e.g., heterosexual vs homosexual people) using differential item functioning should be explored. For instance, it could be that people of different sexual orientation with the same ASB have different probability of selecting one of the categories of an item.

Despite these limitations, it can be concluded that this study has shown favorable evidence of validity of the theoretical model proposed for measurement of ASB in heterosexual young people. The items referring to different sexual behaviors are grouped and distributed in a theoretically coherent manner on the map. In addition, models based on the Item Response Theory, such as Rasch models, allow for a more accurate measure of attitudes toward sexual behaviors than the Classical Test Theory model because they consider the different amount of construct (item difficulty) with which each item contribute to the person measure.

## APPENDIX A: Test of Attitudes toward Sexual Behaviors (TASB)

1. Caressing any intimate part of the body of a casual partner [*Acariciar cualquier zona íntima del cuerpo de una pareja ocasional*].
2. Penile-vaginal sexual intercourse with a casual partner [*Realizar el coito (penetración vaginal) con una pareja ocasional*].
3. Mutual masturbation with a casual partner [*Masturbarse mutuamente con una pareja ocasional*].
4. Oral sex with a casual partner [*Realizar sexo oral con una pareja ocasional*].
5. Anal sex with a casual partner [*Realizar sexo anal con una pareja ocasional*].
6. Send pictures or messages via the internet or a cell phone with sexual content (sexting) to a casual partner [*Enviar imágenes o mensajes a través de internet o móvil (sexting) a una pareja ocasional*].
7. Sex over the internet (cybersex) with a casual partner. [*Tener sexo a través de la red (cibersexo) con una pareja ocasional*].
8. Caressing any intimate part of the body of a steady partner [*Acariciar cualquier zona íntima del cuerpo de una pareja estable*].
9. Penile-vaginal sexual intercourse with a steady partner [*Realizar el coito (penetración vaginal) con una pareja estable*].
10. Mutual masturbation with a steady partner [*Masturbarse mutuamente con una pareja estable*].
11. Oral sex with a steady partner [*Realizar sexo oral con una pareja estable*].
12. Anal sex with a steady partner [*Realizar sexo anal con una pareja estable*].
13. Send pictures or messages via the internet or a cell phone with sexual content (sexting) to a steady partner [*Enviar imágenes o mensajes a través de internet o móvil (sexting) a una pareja estable*].
14. Sex over the internet (cybersex) with a steady partner. [*Tener sexo a través de la red (cibersexo) con una pareja estable*].
15. Solitary masturbation (alone) when a person doesn´t have a steady partner [*Masturbarse en solitario (sin compañía) cuando se está sin pareja*].
16. Solitary masturbation (alone) when a person has a steady partner [*Masturbarse en solitario (sin compañía) cuando se tiene pareja estable*].
17. Having sexual fantasies when a person doesn´t have a steady partner [*Tener fantasías sexuales cuando se está sin pareja*].
18. Having sexual fantasies when a person has a steady partner [*Tener fantasías sexuales cuando se tiene pareja estable*].
19. Reading erotic magazines or books (with sexual content) [*Utilizar revistas o libros eróticos (con contenido sexual)*].
20. Watching erotic movies (for example, showing sexual activities) [*Utilizar películas eróticas (por ejemplo*: *mostrando actividades sexuales)*]
21. Sexual activity with two other persons at the same time (threesome) [*Tener relaciones sexuales con dos personas al mismo tiempo (hacer un trio)*].
22. Sexual activity with a group of persons at the same time (orgy or group sex) [*Tener relaciones sexuales con un grupo de personas al mismo tiempo (hacer una orgía o sexo en grupo)*].

## References

[1] Fisher WA, Byrne D, White LA, Kelley K. Erotophobia-erotophilia as a dimension of personality. J Sex Res. 1988; 25 (1): 123–51. 10.1080/00224498809551448

[2] Hannon R, Hall D, Gonzalez V, Cacciapaglia H. Trueblood Sexual Attitudes Questionnaire In: Fisher TD, Davis CM, Yarber WL, Davis SL, editors. Handbook of Sexuality-Related Measures. 3rd ed New York: Routledge; 2011 p. 68–71.

[3] Wilson SM, Medora NP. Gender Comparisons of College Students' Attitudes toward Sexual Behavior. Adolesc. 1990; 25 (99): 615–27. 2264511

[4] Casey TT. Exploring Sexual Attitudes and Experiences: A Classroom Exercise. Am J Sex Educ. 2011; 6: 306–16. 10.1080/15546128.2011.601961

[5] Eisen M, Zellman GL. Changes in incidence of sexual intercourse of unmarried teenagers following a community-based sex education program. J Sex Res. 1987; 23 (4): 527–33. 10.1080/00224498709551388

[6] Furstenberg FF, Morgan SP, Moore KA, Peterson JL. Race differences in the timing of adolescent intercourse. Am Sociol Rev. 1987; 52 (4): 511–18. 10.2307/2095296

[7] Lehmiller JJ. Sexual Behaviors In Lehmiller JJ, editors. The Psychology of Human Sexuality. Oxford: John Wiley & Sons; 2014 pp. 229–55.

[8] Schuster MA, Bell RM, Kanouse DE. The sexual practices of adolescent virgins: Genital sexual activities of high school students who have never had vaginal intercourse. Am J Public Health. 1996; 86 (11): 1570–76. 10.2105/AJPH.86.11.1570 8916522 PMC1380691

[9] Oliver MB, Hyde JS. Gender differences in sexuality: A meta-analysis. Psychol Bull. 1993; 114 (1): 29–51. 10.1037//0033-2909.114.1.29 8346327

[10] Wells BE, Twenge JM. Changes in young people’s sexual behavior and attitudes, 1943–1999: A cross-temporal meta-analysis. Rev Gen Psychol. 2005; 9 (3): 249–61. 10.1037/1089-2680.9.3.249

[11] Petersen JL, Hyde JS. A meta-analytic review of research on gender differences in sexuality: 1993 to 2007. Psychol Bull. 2010; 136 (1): 21–38. 10.1037/a0017504 20063924

[12] Twenge JM, Sherman RA, Wells BE. Changes in American adults’ sexual behavior and attitudes, 1972–2012. Arch Sex Behav. 2015; 44 (8): 2273–85. 10.1007/s10508-015-0540-2 25940736

[13] Faílde JM, Lameiras M, Bimbela JL. Prácticas sexuales de chicos y chicas españoles de 14–24 años de edad [Sexual behavior in a Spanish sample aged 14 to 24 years old]. Gac Sanit. 2008; 22 (6): 511–19. 10.1590/S0213-91112008000600002 PMID: 19080925 19080925

[14] Rodríguez OR. Relación entre satisfacción sexual, ansiedad y prácticas sexuales [Relationship between sexual satisfaction, anxiety and sexual practices]. Pensam Psicol. 2010; 7 (14): 41–52.

[15] Rosenberger JG, Reece M, Schick V, Herbenick D, Novak DS, Van Der Pol B, et al Sexual behaviors and situational characteristics of most recent male-partnered sexual event among gay and bisexually identified men in the United States. J Sex Med. 2011; 8 (11): 3040–50. 10.1111/j.1743-6109.2011.02438.x 21883941

[16] Hogarth H, Ingham R. Masturbation among young women and associations with sexual health: An exploratory study. J Sex Res. 2009; 46 (6): 558–67. 10.1080/00224490902878993 19350442

[17] Moral de la Rubia J. Predicción de la frecuencia de masturbación en estudiantes universitarios [Prediction of masturbation frequency in college students]. RIP. 2011; 45 (1): 77–86.

[18] Bogaert AF, Visser BA, Pozzebon JA. Gender differences in object of desire self-consciousness sexual fantasies. Arch Sex Behav. 2015; 44 (8): 2299–310. 10.1007/s10508-014-0456-2 25567072

[19] Leitenberg H, Henning K. Sexual fantasy. Psychol Bull. 1995; 117 (3): 469–96. 10.1037/0033-2909.117.3.469 7777650

[20] Boies SC. University students’ uses of and reactions to online sexual information and entertainment: Links to online and offline sexual behavior. Can J Hum Sex. 2002; 11 (2): 77–89.

[21] Goodson P, McCormick D, Evans A. Searching for sexually explicit material on the internet: An exploratory study of college students’ behavior and attitudes. Arch Sex Behav. 2001; 30 (2): 101–117. 10.1023/A:1002724116437 11329723

[22] Muise A, Giang E, Impett EA. Post sex affectionate exchanges promote sexual and relationship satisfaction. Arch Sex Behav. 2014; 43 (7): 1391–402. 10.1007/s10508-014-0305-3 24777441

[23] Heywood W, Patrick K, Smith AM, Pitts MK. Associations between early first sexual intercourse and later sexual and reproductive outcomes: A systematic review of population-based data. Arch Sex Behav. 2015; 44 (3): 531–69. 10.1007/s10508-014-0374-3 25425161

[24] Teva I, Bermúdez MP, Buela-Casal G. Characteristics of sexual behavior in Spanish adolescents. Span J Psychol. 2009; 12 (2): 471–84. 10.1017/S1138741600001852 19899649

[25] Daltos AL, Galambos NL. Affect and sexual behavior in the transition to university. Arch Sex Behav. 2009; 38 (5): 675–87. 10.1007/s10508-008-9401-6 18814022

[26] Fielder RL, Carey MP. Predictors and consequences of sexual ‘‘Hookups” among college students: A short-term prospective study. Arch Sex Behav. 2010; 39: 1105–19. 10.1007/s10508-008-9448-4 19130207 PMC2933280

[27] Kaestle CE, Allen KR. The role of masturbation in healthy sexual development: Perceptions of young adults. Arch Sex Behav. 2011; 40: 983–94. 10.1007/s10508-010-9722-0 21293916

[28] Baldwin JI, Baldwin JD. Heterosexual anal intercourse: An understudied, high-risk sexual behavior. Arch Sex Behav. 2000; 29 (4): 357–73. 10.1023/A:1001918504344 10948725

[29] Daneback K, Cooper A, Månsson SA. An internet study of cybersex participants. Arch Sex Behav. 2005; 34 (3): 321–28. 10.1007/s10508-005-3120-z 15971014

[30] Gámez-Guadix M, Santisteban P, Resett S. Sexting among Spanish adolescents: Prevalence and personality profiles. Psicothema. 2017; 29 (1): 29–34. 10.7334/psicothema2016.222 28126055

[31] Thompson AE, Byres ES. Heterosexual young adults´ interest, attitudes, and experiences related to mixed-gender, multi-person sex. Arch Sex Behav. 2017; 46 (3): 813–22. 10.1007/s10508-016-0699-1 26943139

[32] Rice CE, Lynch CD, Norris AH, Davis JA, Filed KS, Ervin M, et al Group sex and prevalent sexually transmitted infections among men who have sex with men. Arch Sex Behav. 2016; 45 (6): 1411–19. 10.1007/s10508-015-0554-9 26392187

[33] Chambers WC. Oral sex: Varied behaviors and perceptions in a college population. J Sex Res. 2007; 44 (1): 28–42. 10.1080/00224490709336790 17599262

[34] Shaughnessy K, Byers ES. Contextualizing cybersex experience: Heterosexually identified men and women’s desire for and experiences with cybersex with three types of partners. Comput Human Behav. 2014; 32: 178–85. 10.1016/j.chb.2013.12.005

[35] Samimi P, Alderson KG. Sexting among undergraduate students. Comput Human Behav. 2014; 31: 230–41. 10.1016/j.chb.2013.10.027

[36] Cunningham WA, Zelazo PD, Packer DJ, Van Bavel JJ. The iterative reprocessing model: a multilevel framework for attitudes and evaluation. Soc Cogn. 2007; 25 (2): 736–60. 10.1521/soco.2007.25.5.736 PMID: 25168638

[37] Eagly AH, Chaiken S. The Advantages of an Inclusive Definition. Soc Cogn. 2007; 25 (5): 582–602. 10.1521/soco.2007.25.5.582

[38] Fazio RH. Attitudes as object–evaluation associations of varying strength. Soc Cogn. 2007; 25 (5): 603–37. 10.1521/soco.2007.25.5.603 19424447 PMC2677817

[39] Fabrigar LR, MacDonald TK, Wegener DT. The structure of attitudes In Albarracín D D., Johnson BT, Zanna MP, editors. Handbook of attitude and attitude change. Mahwah, NJ: Erlbaum; 2005 pp. 79–124.

[40] Eagly AH, Chaiken S. The psychology of attitudes 1st ed Fort Worth, TX: Harcourt, Brace, Jovanovich; 1993.

[41] Jaccard J, Blanton H. The origins and structure of behavior: Conceptualizing behavior in attitude research In Albarracín D D., Johnson BT, Zanna MP, editors. Handbook of attitude and attitude change. Mahwah, NJ: Erlbaum; 2005 pp. 125–73.

[42] Blanc A, Rojas AJ. Valoración de comportamientos sexuales mediante el método de pares comparados en una muestra española [Assessment of sexual behaviors using the paired-comparisons method in a sample from Spain]. RACC. 2017; 9 (2): 19–33.

[43] Fisher TD, Davis CM, Yarber WL, Davis SL. Handbook of Sexuality-Related Measures (3rd ed). New York: Routledge; 2011.

[44] Wright BD, Masters GN. Rating scale analysis. Chicago: Mesa Press; 1982.

[45] Baghaei P. The Rasch model as a construct validation tool. Rasch Measurement Transactions. 2008; 22: 1145–46.

[46] Linacre JM. Winsteps (computer program and manual). Chicago: Mesa Press; 2007.

[47] Linacre JM. Largest residual correlations for items. Available from: http://www.winsteps.com/winman/table23_99.htm

[48] Engelhard G. Invariant measurement: Using Rasch models in the social, behavioral and health sciences New York: Routledge; 2013.

[49] Weisskirch RS, Delevi R. ‘‘Sexting” and adult romantic attachment. Comput Human Behav. 2011; 27 (5), 1697–701. 10.1016/j.chb.2011.02.008

[50] Centro de Investigaciones Sociológicas (CIS). Actitudes y prácticas sexuales [Attitudes and sexual practices]. 14 Jan 2018. Available from: http://www.cis.es/cis/opencms/-Archivos/Marginales/2720_2739/2738/ES2738mar.pdf Cited 15 Jan 2017

[51] Wittes B, Poplin C, Jurecic Q, Spera C. Sextortion: Cybersecurity, teenagers, and remote sexual assault. Center for Technology Innovation at Brookings. 5 2016 Available from: http://www.brookings.edu/~/media/Research/Files/Reports/2016/05/sextortion/sextortion1.pdf?la=en Cited 15 Jan 2017

